# Influence of Electrospinning Parameters on the Morphology of Electrospun Poly(3-hydroxybutyrate-co-3-hydroxyvalerate) Fibrous Membranes and Their Application as Potential Air Filtration Materials

**DOI:** 10.3390/polym16010154

**Published:** 2024-01-04

**Authors:** Yaohui Liu, Yanming Wang, Cheng-Hao Lee, Chi-Wai Kan, Xiaoying Lu

**Affiliations:** 1 Faculty of Science and Technology, Technological and Higher Education Institute of Hong Kong, Tsing Yi, New Territories, Hong Kong, China; amyymwang@vtc.edu.hk (Y.W.); xylu@thei.edu.hk (X.L.); 2 School of Fashion and Textiles, The Hong Kong Polytechnic University, Hung Hom, Kowloon, Hong Kong, China; chenghao.lee@polyu.edu.hk

**Keywords:** PHA, PHBV, electrospinning, fibrous membrane, air filtration, biodegradable

## Abstract

A large number of non-degradable materials have severely damaged the ecological environment. Now, people are increasingly pursuing the use of environmentally friendly materials to replace traditional chemical materials. Polyhydroxyalkonates (PHAs) are receiving increasing attention because of the unique biodegradability and biocompatibility they offer. However, the applications of PHAs are still limited due to high production costs and insufficient study. This project examines the optimal electrospinning parameters for the production of PHA-based fibrous membranes for air filtration. A common biodegradable polyester, Poly(3-hydroxybutyrate-co-3-hydroxyvalerate) (PHBV), was electrospun into a nanofibrous membrane with a well-controlled surface microstructure. In order to produce smooth, bead-free fibers with micron-scale diameters, the effect of the process parameters (applied electric field, solution flow rate, inner diameter of hollow needle, and polymer concentration) on the electrospun fiber microstructure was optimized. The well-defined fibrous structure was optimized at an applied electric field of 20 kV, flow rate of 0.5 mL/h, solution concentration of 12 wt.%, and needle inner diameter of 0.21 mm. The morphology of the electrospun PHBV fibrous membrane was observed by scanning electron microscopy (SEM). Fourier transform infrared (FTIR) and Raman spectroscopy were used to explore the chemical signatures and phases of the electrospun PHBV nanofiber. The ball burst strength (BBS) was measured to assess the mechanical strength of the membrane. The small pore size of the nanofiber membranes ensured they had good application prospects in the field of air filtration. The particle filtration efficiency (PFE) of the optimized electrospun PHBV fibrous membrane was above 98% at standard atmospheric pressure.

## 1. Introduction

In recent years, extensive damage has been inflicted upon the ecological environment due to the production of substantial quantities of non-degradable materials associated with the fast-paced expansion of the chemical industry. Owing to the long-term economic benefits of environmental protection, there has been increasing interest in substituting traditional chemical materials with biodegradable alternatives [1,2,3,4,5]. Polyhydroxyalkanoates (PHAs) and silk have gained significant attention due to their special biodegradability and biocompatibility properties [6,7,8,9].

**Figure 1:**
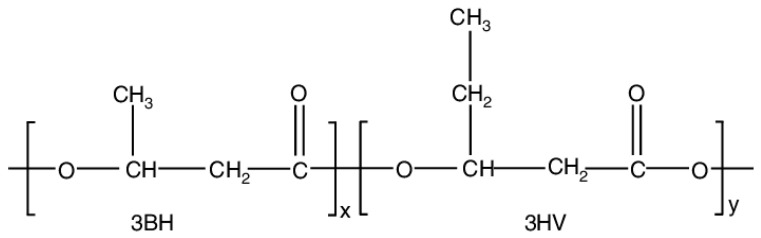
Chemical structure of Poly(3-hydroxybutyrate-co-3-hydroxyvalerate) (PHBV).

Research on PHAs has been predominantly focused on melt spinning and electrospinning. PHAs’ melt spinning process is still at the laboratory stage due to the persistence of crystallization and thermal stability imperfections [10,11]. Poly(3-hydroxybutyrate-co-3-hydroxyvalerate) (PHBV) has seen significant exploration within the PHA family (Figure 1). PHBV is an environmentally friendly thermoplastic polyester that can be produced by certain types of bacteria in the presence of excessive nitrogen and carbon sources, including cellulose, glucose, and sugar cane molasses [12,13,14]. PHBV has good oxygen permeability, mechanical strength and nontoxic. The ultimate degradation product of PHBV is (R)-3-hydroxybutyric acid, which is a normal constituent of human blood. Therefore, PHBV has been extensively explored as a biomaterial for both in vitro and in vivo studies [15,16,17]. Because of its outstanding biocompatibility and biodegradability, PHBV nanofiber membranes are typically employed in the fields of numerous biomedical devices, including sutures, prosthetic devices, drug delivery systems, and surgical applications, although their application in air filtration has been comparatively less explored [18,19]. Meanwhile, PHBV possesses a relatively high level of crystallinity, which provides it with a high mechanical strength, and an excellent level of biocompatibility, making it a suitable material for use in biodegradable air filters and personal protective equipment (PPE). This is due to its excellent biosafety properties [20,21]. 

Electrospinning is a low-cost and versatile technology that allows for the production of nanofibers from a wide range of polymers [22,23]. Some polymers, such as polyamide (PA) [24], polyethylene terephthalate (PET) [25], polyacrylonitrile (PAN) [26], polylactic acid (PLA) [27], and polyvinyl alcohol [28,29], have been extensively electrospun for air filtration purposes and are often used for meltblown or spunbond nonwoven textiles [30]. Electrospun nanofiber membranes possess diameters of several hundred nanometers, an extensive surface-area-to-volume ratio, and small interconnected pores [31]. Consequently, they are an efficient filtration medium, capable of capturing fine particles (ranging from 300 to 500 nm), volatile organic gases, and bacteria [32]. Nanofiber diameters and the packing structure of electrospun membranes result in a superior performance compared with traditional meltblown nonwoven filters made of microfibers [33]. As a result, nanofibers with a weight of approximately one gram per square meter may be equally effective as a melt blown filter with a weight of several tens of grams per square meter. Consequently, electrospun nanofiber membranes have a low volume, high efficiency in capturing particles with a size smaller than 2.5 µm (PM 2.5), and decreased breathing restriction (pressure drop) [34,35]. The electrospinning process offers an advantage in creating composite membranes, which consist of a layer of electrospun nanofibers over a robust nonwoven microfiber structure such as meltblown or spunbond procedures. This technique has been demonstrated in previous research [36]. 

This study examined the influence of electrospinning parameters on the morphology of electrospun poly(3-hydroxy-butyrate-co-3-hydroxyvalerate) (PHBV) fibers using chloroform as the dispersing solvent, which possess a low toxicity and residue. The objective was to optimize the electrospun PHBV membrane, and, subsequently, based on the experimental conclusion, the potential application of air filtration may be further explored. It is important to note that a comprehensive assessment of electrospun PHBV membranes and their effectiveness in filtering particulate matter of varying sizes has not been previously documented, to the best of our knowledge [37,38,39].

## 2. Materials and Methods

### 2.1. Electrospinning Process

**Figure 2:**
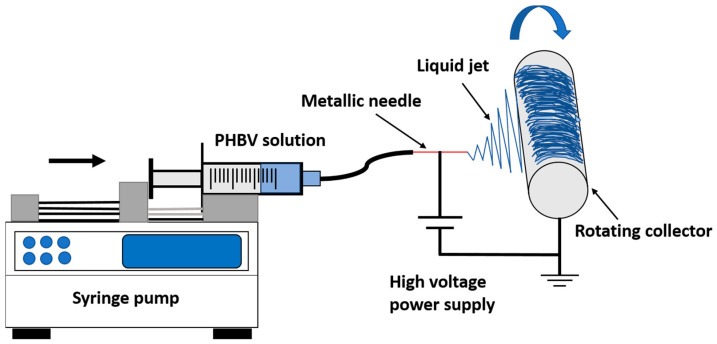
Schematic diagram of the electrospinning process.

Poly(3-hydroxybutyrate-co-3-hydroxyvalerate) (PHBV), HB:HV = 1:1, MW = 30,000 kDa (Cheng Jin Plastics Co., Ltd., Dongguan, Guangdong, China) was prepared in a homogeneous solution with a concentration 7–15 wt.% by dissolving it in chloroform (GR grade, 99.8% purity, RCI Labscan Ltd., Bangkok, Thailand). Prior to electrospinning, the solution was filled with a 50 mL glass syringe with a metal luer lock tip (SAMCOTM, Weymouth, UK). It was continuously pushed by a syringe pump to ensure steady flow rates of 0.5, 1, 3, and 5 mL/h as the effect of different flow rates needed to be examined. To investigate the effect of applied voltage (9–20 kV), solution concentration (12–15 wt.%), flow rate (0.5–5 mL/h), and tip inner diameter (ID: 0.16–0.5 mm), the distance between the needle tip and cylindrical collector was fixed at 16 cm. The polymer solutions were filled in a standard 50 mL syringe (SAMCO Co., Ltd., Nuneaton, UK) containing the polymer solution. The polymer solution was driven out of the syringe using an NE-300 single syringe pump (Model TL-F6, Tong Li Tech. Co., Ltd., Shenzhen, China). As illustrated in Figure 2, a metal syringe needle (SAMCO Co., Ltd., Nuneaton, UK) was attached to the syringe. These needles were ground down to produce a blunt tip, allowing for the formation of a more stable Taylor cone prior to use. The needle tip and rotating cylindrical collector were connected to a high voltage power supply (Model TL-Pro, Tong Li Tech Co., Ltd., Shenzhen, China), which can provide positive DC voltage up to 30 kV. The applied voltage was set at 20 kV and the flow rate and concentration were kept constant for effect studies. A flow rate of 0.5 mL/h was used in order to create a stable system whereby the fluid forced into droplet was equivalent to the average rate at which the fluid was carried away by the jet. 

### 2.2. Characterization

Electrospun fiber were coated with a thin gold layer using a sputtering coater (E-1010, Hitachi Co., Tokyo, Japan) and their morphology were analyzed using scanning electron microscopy (SEM) (TM 3000 Table Top SEM, Hitachi Co., Tokyo, Japan) with an accelerating voltage of 15 kV. The diameter of the fibers was measured using SEM image (1000× magnification) and the Image J version 1.54h software.

The diffuse reflection IR measurements were carried out by employing a Perkin Elmer Spectrum 100 FTIR spectrometer prepared with a 3″ high reflectivity gold-coated integrating sphere (PIKE Mid-IR IntegratIR™, Barrington, NJ, USA). The spectral detector was a low-noise, high speed liquid-nitrogen-cooled mercury−cadmium−telluride (MCT) connected to the external detector port of the spectrometer. The spectral resolution was 4 cm^−1^.

The Raman spectrum was recorded on a BaySpec Nomadic™ Raman Microscope equipped with a confocal microscope (San Jose, CA, USA). Visible excitation was generated by a diode-pumped solid-state continuous wave laser (532 nm and 50 mW). The acquisition time was 1 s and 3 scans were required for each spectrum. Raman spectra were recorded with a laser spot size of 200 μm diameter. Therefore, the intensity signals over this area represent the spectral features of the material. No significant differences were found in recorded the spectra of various film regions.

The mechanical properties of the electrospun PBHV fibrous membrane and ASTM F2100 level 1 mask [40] were investigated by ball burst testing using an INSTRON 4411 Tensile Compression Testing Machine (Instron Corporation, Norwood, MA, USA). A 13 cm × 12 cm electrospun fibrous membrane with a thickness 0.14 mm ± 0.02 mm and level 1 mask were clamped between two metal rings and held tightly for further testing according to ASTM standard D3787-07 [41]. The metal ring was lined with sandpaper throughout the ball burst testing process in order to prevent the mesh from slipping. The data collected from each sample were recorded in load (Newtons) vs. elongation (mm).

The efficiency of filtration of PMs was tested by a PMs laser scattering detector (HYX-MS600-6, Shenzhen, China). The air flow rate was 85 L/min, the effective test area and the air resistance were 3.2 cm^2^ and 12.5 Pa, respectively.

## 3. Results

### 3.1. Effect of Applied Voltage

**Figure 3:**
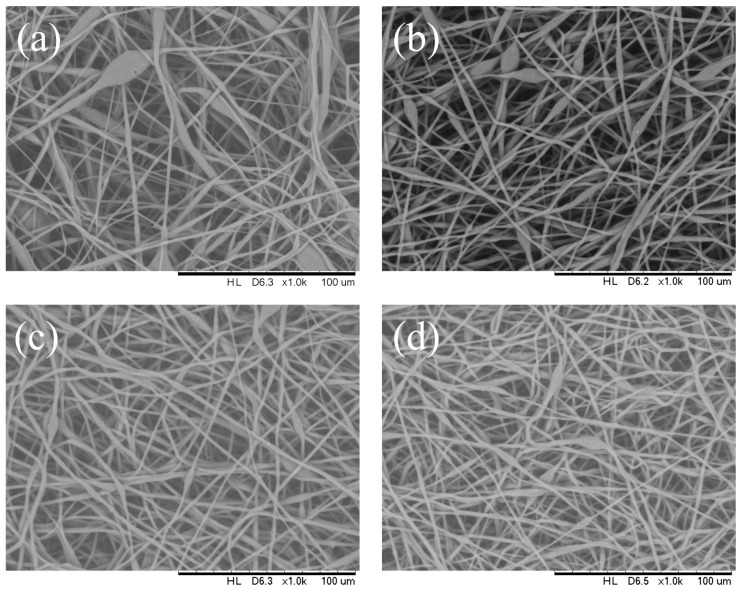
SEM images of the electrospun PHBV nanofibers at an applied voltage of (**a**) 9 kV, (**b**) 12 kV, (**c**) 18 kV, and (**d**) 20 kV.

Figure 3 shows the morphological difference in electrospun PHB filaments that were the outcome from different applied voltages (9 kV to 20 kV). In this article, we explored the thickness of the bead toward the path opposite to the fiber hub. Under electrospinning conditions of 14 wt.% PHBV solution, flow rate of 1 mL/h, needle inner diameter of 0.33 mm, fibers with porous beads were produced with an average bead size of 8 to 16 μm and a mean fiber diameter of 2 μm at an applied voltage of 9 kV. The number of beads observed decreased significantly and the bead size range was observed to be reduced to 5–7 μm under 18 kV applied voltage, while the fiber diameter remained roughly the same. A further expansion in applied voltage (20 kV) brought about a more modest bead size range of 4–5 μm and a mean fiber diameter was approximately 1 μm (Table 1). For a 14 wt.% PHBV solution at a flow rate of 1 mL/h, a voltage at 20 kV led to well distributed fiber with a mean diameter of 1 μm. Consequently, it was concluded that the bead-on-string morphology tended to be suppressed at higher voltages.

The distribution of the fiber diameter became narrow as the voltage increased. The bead structure almost vanished when the applied voltage was 20 kV. However, the surface morphology of the fibers was not affected when the voltage increased up to 20 kV.

### 3.2. Effect of Solution Flow Rate

**Figure 4:**
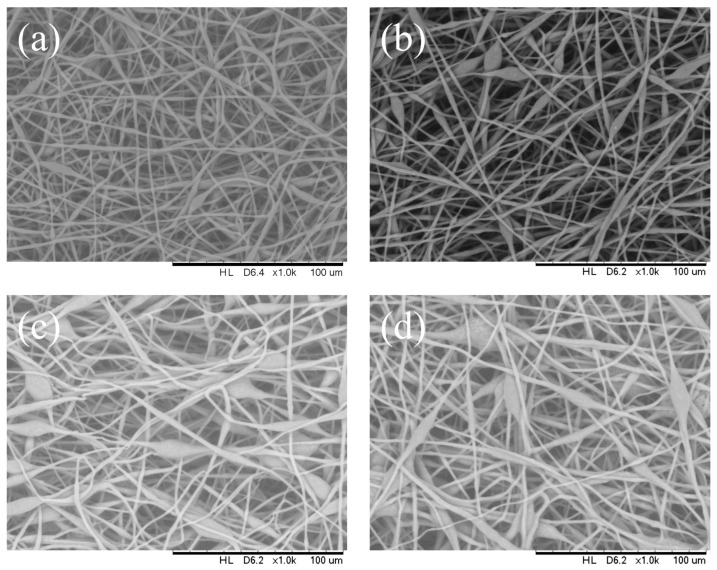
SEM images of the electrospun PHBV nanofibers at a solution injection flow rate of (**a**) 0.5 mL/h, (**b**) 1 mL/h, (**c**) 3 mL/h, and (**d**) 5 mL/h.

The solution flow rate was controlled by the syringe pump to determine its effect on the fiber microstructure at varying feed rates of 0.5, 1, 3, and 5 mL/h (solution concentration 14 wt.%, applied voltage 12 kV, needle inner diameter of 0.33 mm, collector distance 16 cm, and temperature of 25 °C). As shown in Figure 4, the fiber diameter in the bead-on-string structure increased when the flow rate was increased from 0.5 to 5 mL/h. A continuous fiber structure was observed at a flow rate of 1 mL/h or less. As shown in Figure 4, at a flow rate of 5 mL/h, bead-on-string structures were observed from the PHBV membrane with a mean diameter ranging from 7 µm to 16 µm and, in particular, the PHBV membrane obtained from the solution at a flow rate above 3 mL/h had significant porous structures on the bead surface. The mean fiber diameter was observed from 2 to 4.6 μm as the flow rate increased from 0.5 to 5 mL/h (Table 2). The fiber ‘s morphology shifted from fiber to bead-on-string as the flow rate increased, with a noticeable change in the bead dimension. From the results, it can be concluded that the optimum flowrate of the PHB solution for well-defined uniform fiber with a minimal number of beads should be maintained at 1 mL/h or below.

It was observed that increasing the solution flow rate from 0.5 up to 5 mL/h decreased the mean fiber diameter. On the other hand, the fiber diameter distribution was very similar for the samples collected at different flow rates. It is generally expected that the decrease in fiber diameter and increase in bead diameter with an increase in flow rate is due to the larger volume of solution that is ejected from the needle in a short period. This increase in volume drawn from the needle tip generates a superfluous solution with a high surface tension and the moving jet is not able to stretch completely. Consequently, beads distributed along the fibers are generated due to surface tension and axisymmetric jet instabilities [4].

### 3.3. Effect of Capillary Inner Diameter of Needle 

**Figure 5:**
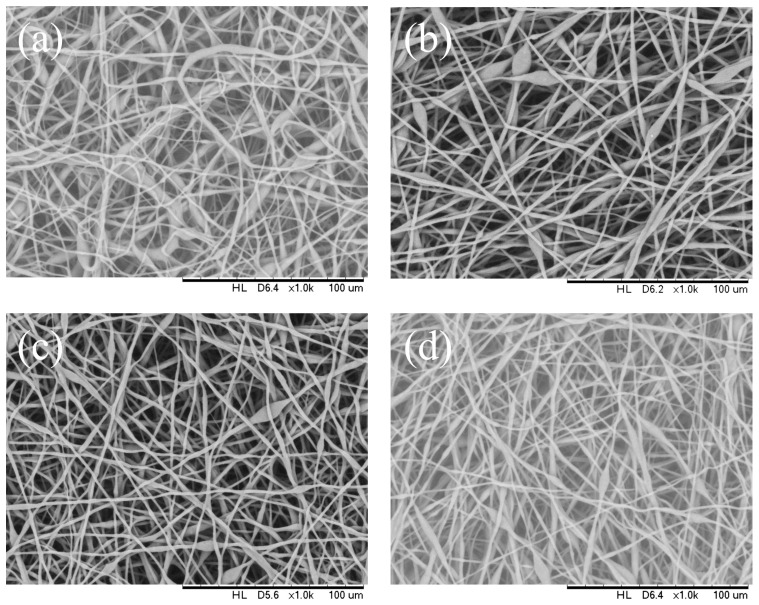
SEM images of the electrospun PHBV nanofibers under the effect of a capillary inner diameter of (**a**) 0.5 mm, (**b**) 0.33 mm, (**c**) 0.21 mm, and (**d**) 0.16 mm.

Morphologies of electrospun PHBV fibers from needles of various capillary inner diameters are shown in Figure 5. The inner diameter varied from 0.16 mm to 0.5 mm. All of the other parameters were kept unchanged (solution concentration 14 wt.%, flow rate 1.0 mL/h, and applied voltage 12 kV). These SEM micrographs illustrate that the fiber diameter generally increases with increasing the needle inner diameter. Using positive voltage electrospinning, the bead diameter ranged from 4 to 6 μm when the 0.5 mm ID needle was used. The bead diameter distribution for the 0.33 mm ID needle was found to have shifted to a mean size ranging from 4 μm to 8.5 μm. Electrospun fibers with only a small percentage of bead-on-string structures (size range from 3 to 6 μm) were observed when the 0.21 mm ID needle was used (Figure 5a–c). In other words, the mean fiber diameter was observed from 2.55 to 1.83 μm as the inner needle diameter changed from 0.5 mm to 0.16 mm (Table 3). As indicated in Figure 5d, although the introduction of a 0.16 mm inner diameter needle could effectively suppress the formation of the bead-on-string structure, the internal pressure exerted by the solution on the silicone tube or the syringe was relatively high when the orifice of the needle was small. The high pressure exerted could eventually damage the silicone tube or the syringe pump; hence, it was not suitable for the production of a large area membrane via the electrospinning process [42,43,44]. It would eventually downgrade both the fiber quality and production rate if the pressure was reduced by decreasing the solution feeding rate. The use of a syringe needle with ID 0.5 mm or larger was also problematic because the polymer solution was readily exposed to air at the large needle pinhole. The solvent would then evaporate easily and quickly and the polymer solution pendent droplet would solidify shortly. The solidified polymer could induce blockage of the needle and the process could be terminated shortly after electrospinning started.

### 3.4. Effect of Solution Concentration

**Figure 6:**
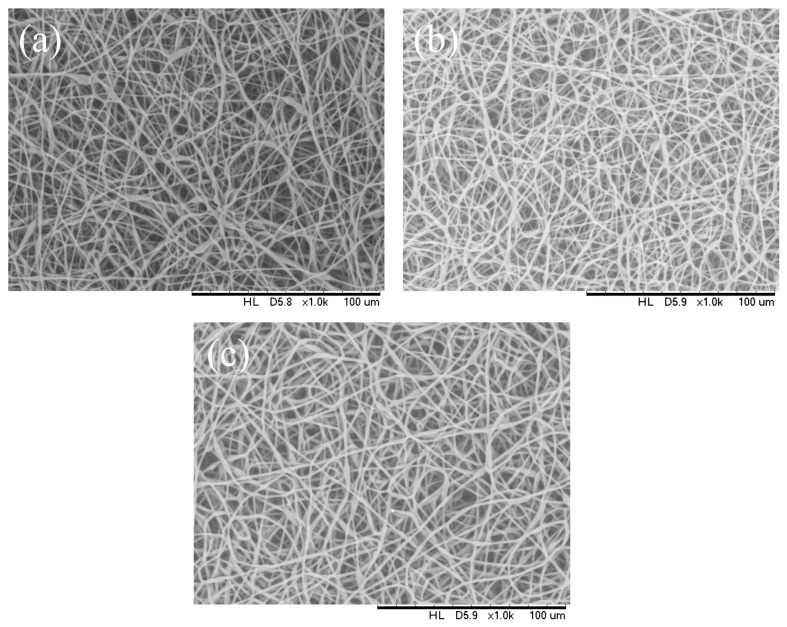
SEM images of the electrospun PHBV nanofibers prepared at (**a**) 12 wt.%, (**b**) 13 wt.%, and (**c**) 14 wt.%. of PHBV in chloroform.

The concentration of the PHBV solution varied from 12 to 15 wt.%. All of the other parameters were kept constant (flow rate 0.5 mL/h, applied voltage of 20 kV, capillary needle inner diameter of 0.16 mm, tip-collector distance of 16 cm, and temperature of 25 °C). The electrospun fibers are shown in Figure 6. It can be found that the fiber structure was well-defined with a similar mean diameter of about 2.55 ± 0.03 μm as the polymer concentration increased from 12 wt.% to 14 wt.% (Table 4). With the polymer fraction being lower or higher than the critical point concentration, a bead-on-string structure was formed in the metastable region under nucleation and growth processes, located between the binodal and spinodal curves [34,45,46].

However, the phase separation at a polymer concentration higher than 14 wt.% was difficult and, therefore, it was difficult for a fiber-like structure to be formed due to high viscosity. As the polymer chain mobility was inversely proportional to the solution concentration; a higher weight percentage of the polymer favored the accumulation of a solidified polymer at the pinhole position of the needle. 

### 3.5. ATR-FTIR Spectroscopy of Electrospun PHBV Fiber Membrane

**Figure 7:**
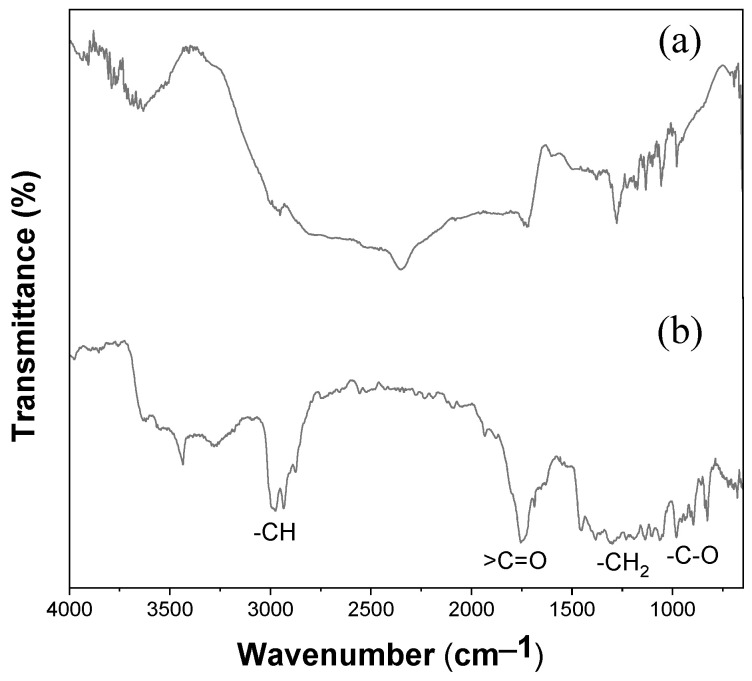
FTIR spectrum of PHBV (**a**) before electrospinning and (**b**) after electrospinning.

The electrospun PHBV membrane was analyzed by FTIR spectroscopy to gain insights into its chemical structure; the molecular confirmation results are illustrated in Figure 7. The C–C stretching band occurred at 977 cm^−1^ [47,48]. The C–O stretching bands of the saturated ester linkage were observed between 1054 and 1129 cm^−1^ and from 1226 to 1275 cm^−1^ [47,48,49,50]. The band at 1179 cm^−1^ was assigned to the C–O–C stretching of the amorphous PHBV [51]. The band at 1720 cm^−1^ (highest intensity) was assigned to a C=O stretching of an ester group in PHBV [47,49,52]. The C–H stretching bands of the methyl (–CH_3_) and methylene (–CH_2_) groups appeared at 2975 cm^−1^ and 2933 cm^−1^, respectively [48,49,53]. 

The absorption peaks at 1383 and 1449 cm^−1^ corresponded to the respective stretching and bending mode of the methyl (–CH_3_) group. The peaks at 2930, 1729, and 3430 cm^−1^ were the characteristic peaks of methine (–CH), –carbonyl (C=O), and hydroxyl (–OH) groups, respectively [51,53]. 

PHBV was used in the electrospinning process are dissolved in chloroform and the fabricated fibers could retain a trace amount of solvent. It is important to develop fast, easy-to-use methods to assess solvent retention and facilitate the removal of the residual solvent. Chloroform is highly volatile during the electrospinning process and there will be no significant solvent remaining in the samples [38]. The qualitative identification of the residual solvent via Fourier transform infrared spectroscopy (FTIR) provided an estimation of residual solvent in electrospun fibers. The electrospun fibers treated at 60 °C for 1 h should efficiently remove the retained chloroform with subsequent FTIR analysis. Figure 7 shows the FTIR spectra of PHBV samples before and after electrospinning for comparison. No signature of chloroform peaks was observed for the fabricated PHBV fibrous membrane after the electrospinning process. Furthermore, characteristic peaks for chloroform, C-H stretching at 3019 cm^−1^, C-H bending at 1200 cm^−1^ and C-Cl stretching at 757 cm^−1^, were not identified, indicating no residual solvents following drying treatment in a 60 °C oven.

### 3.6. Raman Spectroscopy of Electrospun PHBV Fiber Membrane

**Figure 8:**
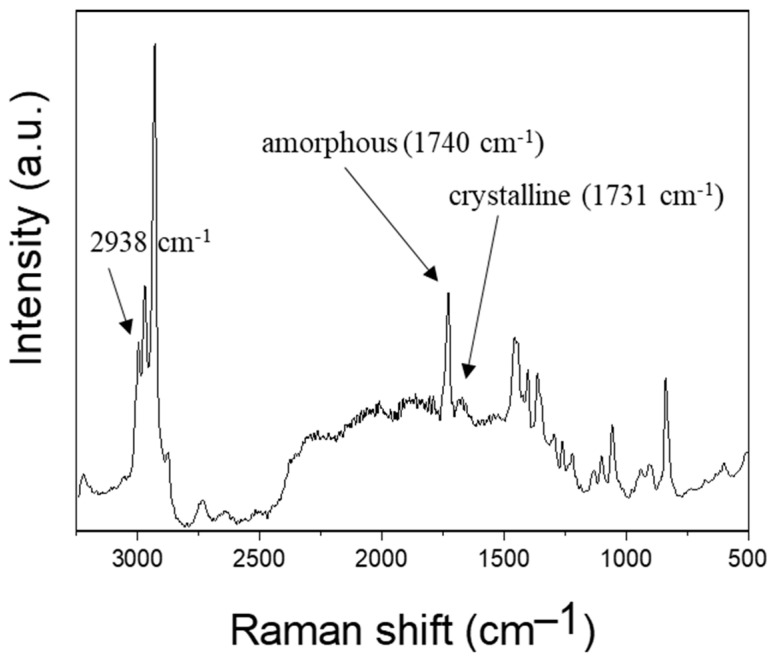
Raman spectra of PHBV electrospun fibrous membrane.

As indicated from the Raman spectrum in Figure 8, the crystalline phase of PHBV was identified with the peaks at 1725 and 1731 cm^−1^, while the amorphous phase could be characterized from the Raman peak at 1740 cm^−1^.

Based on the chemical structure of PHBV, it can be deduced that the bands associated with C-H stretching should govern the Raman spectra in terms of composition. HV and HB units each have one carbonyl group; however, HV units have two methylene groups whereas HB units only have one [54]. The percentage of amount (%) of HV in the polymer samples was the ratio between the peak area in the region of 2805–3070 cm^−1^ with a peak at 2938 cm^−1^ (νC–H) and a sharp peak at 1740 cm^−1^ (νC=O). The relationship between A2938 and A1740 was proportionate to the sample’s HV%. The FTIR spectra of PHBV with samples containing up to 47% of HV units showed a similar profile in the previous study [20]. It should be emphasized that in order to create a trustworthy curve for the quantitative analysis of HV using the Raman spectroscopic approach, a greater number of samples with various HV/HB contents are required [55].

The percentage of crystallization in the electrospun fibers is a critical factor that determines the physical properties of the fibers. As shown in Table 2, the electrospinning parameters may affect the percentage of the crystalline phase. By controlling these parameters, it is possible to tailor the crystallinity of the electrospun fibers to meet specific application requirements. The percentage of crystallinity in the electrospun fibrous membrane is slightly dependent of the process parameters and the range is from 41.7 to 46.4%. 

As the polymer concentration changed from 12 wt.% to 13 wt.%, the percentage of crystallinity was slightly decreased due to random chain entanglements in amorphous state. For an applied voltage switched from 20 kV to 9 kV, the stretching of the polymer jet decreased, which led to a slightly lower crystallinity. From the flow rate effect study, a slower flow rate (0.5 mL/h) led to a higher percentage of crystallinity of 46.4% compared with a flow rate of 5 mL/h due to a longer retention time under electric field exposure. When the needle inner diameter was adjusted from 0.21 mm to 0.5 mm, clogging tended to occur at the needle tip in a very short period of time before sufficient fibers could be collected. Regarding the effect of the needle diameter on electrospun fibers, a larger needle inner diameter resulted in the formation of a larger Taylor cone at the initial electrospinning stage, which facilitated significant exposure of the polymer solution to the air and unstable polymer jet with more disordered chain arrangement. 

### 3.7. Ball Burst Strength (BBS)

**Figure 9:**
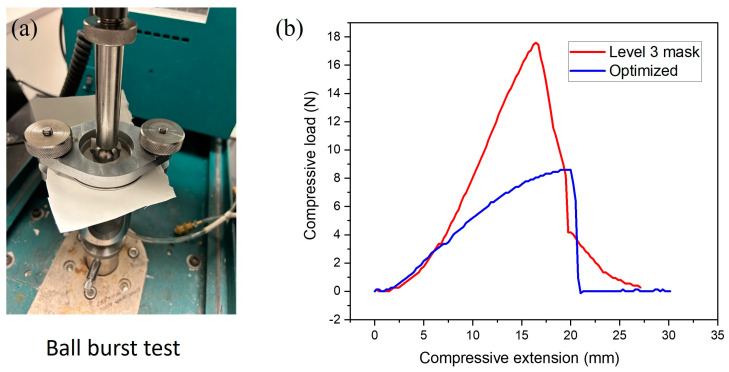
(**a**) The appearance of ball burst apparatus used to examine the mechanical strength and stiffness of the fabric. (**b**) Ball burst (load−elongation) curves of the PHBV fibrous membrane and ASTM F2100 level 3 surgical mask.

The ball burst strength and elongation findings are presented in Figure 9 and Table 5. The PHBV membrane had a maximum compressive load of 8.6 N, which was close to 17.59 N (the load value of the ASTM F2100 level 3 mask). The elongation at burst of the level 3 mask was 16.44 mm and the PHBV fibrous membrane had a comparatively higher elongation at burst of 19.99 mm. Under a similar thickness, the electrospun PHBV fibrous membrane clearly exhibited an acceptable performance compared to market-available surgical masks. The PHBV fibrous membrane showed a higher elongation at burst due to the uneven fiber structures produced by the melt-blown process. When force was applied, the electrospun fiber structure partially burst and the elongation could sustain the level 3 mask slightly longer with more uniform fiber diameters [56,57].

### 3.8. Filtration Efficiency

**Figure 10:**
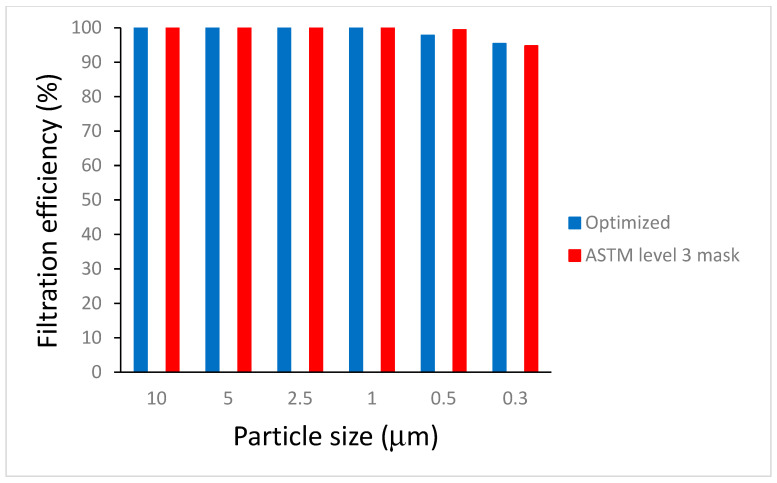
Particle filtration efficiency of electrospun PHBV membrane (PFE: 98.9%) and ASTM F2100 level 3 mask (PFE:99.0%).

Considering their practical application, the electrospun PHBV nanofiber membranes needed a reference fabric or membrane for the evaluation of the filtration property. In this paper, the commercial non-biodegradable PP melt-blown membrane was selected as the reference fabric. The filtration efficiencies of all membranes were tested with average thickness 0.14 mm with standard deviation of 0.04 mm, depending on the spinning time. Table 6 shows the filtration efficiency of the electrospun PHBV fibrous membrane under various parameters. From the filtration efficiencies of various sizes of particulate matter (Figure 10), the particle filtration efficiency of the ASTM F2100 level 1 mask fabric (polypropylene non-woven fibrous membrane) was found to be 99.0%, which fulfilled the filtration requirements. However, the filtration efficiency of the electrospun PHBV membrane under optimized parameters (20 kV, 0.5 mL/h, 12 wt.% concentration, and capillary inner diameter of 0.21 mm) was 98.9%. According to GB 19083-2010 [58], in the case of a gas flow rate of 85 L/min, the inhalation resistance of the mask was less than 343.2 Pa. The electrospun PHBV membrane that exhibited an air resistance of 12.5 Pa was obviously considered to be an effective filter protection material [59,60,61,62].

The mean pore diameter on the surface of straight fibers was estimated to be in the submicron range (less than 1 μm), which was in agreement with the particle size. Therefore, the pores on the fiber surface were able to trap testing particles with a size ranging from 0.3 to 10 µm. On the other hand, the specific surface area of the filter was highly correlated with the pore size. A filter made of small-diameter fibers would have a high particle collection efficiency based on the particle diffusion mechanism [59]. However, a filter with a smaller pore size at the inner layer would strongly influence the direct collection of larger particles. Based on the molecular sieve effect, the size of the particle should be equal to or larger than the mean pore size of the fibrous membrane in order for effective particle capturing. Additionally, a rough fiber surface can provide a significant friction coefficient that facilitates particle collection [63].

The pore size and packing density of a fibrous structure are somewhat influenced by the bead-on-string morphology of the fiber. Meanwhile, the filtration performance is also influenced by this fiber architecture. A steady flow state in the membrane medium with a bead-on-string morphology is quite difficult to create, which also affects the diffusion kinetics of the aerosol particles [60,61]. According to the sieve effect, the filtration performance for three aerosol particle sizes through a membrane structure with bead-on-string fibers is suitable for trapping particles larger particles and unsuitable for filtering particles smaller than 2.5 µm [62,64].

## 4. Conclusions

This paper describes an investigation on the effect of key parameters (applied voltage, flow rate, needle inner diameter, and polymer concentration) on the surface microstructures. The surface bead-on-string structures were associated with a low weight percentage of PHBV in the solution, high flow rate, and low applied voltage. However, the optimized parameters for the smooth well-defined electrospun fiber membrane were flow rate 0.5–1 mL/h, 12 wt.% PHB concentration, needle inner diameter of 0.21 mm, 16 cm collect distance, and applied voltage 20 kV.

The parameters of applied voltage, solution flow rate, and capillary inner diameter had a significant effect on the surface structure. However, the parameters of the solution concentration played a decisive role in the formation of the fiber microstructure. The PHBV electrospun matrix was used as the potential filtration membrane compared with the commercial air filtration mask.

## Figures and Tables

**Table 1:** Effect of applied voltage on fiber diameter range under electrospinning conditions (solution concentration of 14 wt.%, flow rate at 1 mL/h, and needle inner diameter of 0.33 mm).

Applied voltage (kV)	9	12	18	20
Mean fiber diameter (μm)	2	1.4	1.26	1

**Table 2:** Effect of solution flow rate on fiber diameter range under electrospinning conditions (solution concentration of 14 wt.%, applied voltage at 12 kV, needle inner diameter of 0.33 mm).

Injection flow rate (mL/h)	0.5	1	3	5
Mean fiber diameter (μm)	2	2.57	3.6	4.6

**Table 3:** Effect of the capillary inner diameter of the needle on the fiber diameter range under elec trospinning conditions (solution concentration of 14 wt.%, flow rate of 1.0 mL/h, and applied voltage of 12 kV).

Capillary inner diameter (mm)	0.5	0.33	0.21	0.16
Mean fiber diameter (μm)	2.55	2.47	2.36	1.83

**Table 4:** Effect of the solution concentration on the fiber diameter range under electrospinning conditions (flow rate at 0.5 mL/h, applied voltage of 20 kV, and capillary needle inner diameter of 0.16 mm).

PHBV concentration (wt.%)	12	13	14
Mean fiber diameter (μm)	2.55	2.53	2.54

**Table 5:** Ball burst strength of electrospun PHBV membrane and ASTM F2100 level 3 PP mask.

	Maximum Compressive Load (N)	Compression Extension at Burst (mm)
PHBV membrane	8.6	19.99
ASTM F2100 level 3 mask	17.59	16.44

**Table 6:** Effect of electrospinning parameters on surface morphology and particle filtration efficiency (PFE).

Parameters		SEM Image of Electrospun Fiber Membrane	Particle Filtration Efficiency (PFE)	% Crystallinity
0.5 mL/h 20 kV 0.21 mm	12 wt.%	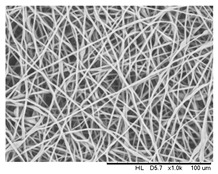	98.9%	46.4
	13 wt.%	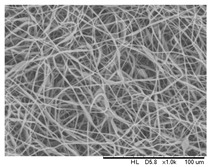	96.1%	43.5
12 wt.% 20 kV 0.21 mm	5 mL/h	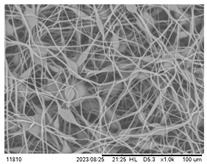	91.1 %	41.7
12 wt.% 0.5 mL/h 0.21 mm	9 kV	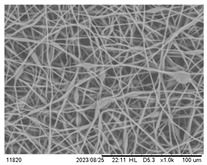	94.2%	45.4
12 wt.% 0.5 mL/h 20 kV	0.5 mm	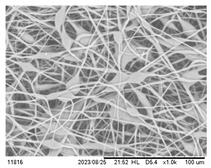	93.6%	42.2

## Data Availability

Data are contained within the article.
